# Level of adherence to option B plus PMTCT and associated factors among HIV positive pregnant and lactating women in public health facilities of Hawassa city, Southern Ethiopia

**DOI:** 10.1371/journal.pone.0255808

**Published:** 2021-08-05

**Authors:** Samuel Abdisa, Zelalem Tenaw

**Affiliations:** Department of Midwifery, College of Medicine and Health Sciences, Hawassa University, Hawassa, Ethiopia; University of Mississippi Medical Center, UNITED STATES

## Abstract

**Background:**

Adherence to antiretroviral therapy is very essential to achieve a great outcome of drugs via suppressing viral load, preventing multidrug resistance, and reducing mother to a child transmission rate of the Human Immune Virus.

**Objective:**

This study aimed to assess the level of adherence to option B plus PMTCT and associated factors among HIV Positive pregnant and lactating women in public health facilities of Hawassa city, Southern Ethiopia, 2020 G.C.

**Methods:**

Institution-based cross-sectional study was done on 254 HIV-positive pregnant and lactating women attending the prevention of mother-to-child transmission (PMTCT) follow-up. Participants were selected by simple random sampling. Data collected through a structured interviewer-administered questionnaire were cleaned and entered into Epi-data 3.1 and exported to SPSS 20 for statistical analysis. Descriptive analysis was done. Bivariable and multivariable logistic regressions were done to measure the strength of association between independent and dependent variables using the odds ratio and 95% of confidence interval. A p-value <0.05 was taken as statistically significant.

**Result:**

The overall adherence level to option B+ was 224 (88.2%). Respondents in age group of ≤ 25 [AOR = 0.12, 95% CI (0.03, 0.42)], with no formal education [AOR = 0.12, 95% CI (0.03, 0.51)], experienced drug side effects [AOR = 0.11, 95% CI (0.04, 0.32)], have good knowledge of PMTCT [AOR = 3.6, 95% CI (1.16, 11.3)], and get support from partner/family [AOR = 4.5, 95% CI (1.62, 12.4)] were identified associated factors with adherence level.

**Conclusion:**

The level of adherence to option B plus PMTCT was 88.2% which is suboptimal. Ages, educational level, knowledge on PMTCT, getting support from partner/family, and drug side effect were significantly associated with adherence. Therefore, educating and counseling on the service of PMTCT to improve their knowledge and encouraging partner/family involvement in care are mandatory to achieve the standard adherence level.

## Introduction

Globally, mother-to-child transmission (MTCT) accounts for over 90% of 180,000 new pediatric HIV infections in 2017 [1]. To avert this way of transmission, different options of PMTCT programs like option A, option B, and option B plus have been developed and implementing. Option B plus PMTCT is a newly proposed option with the feature of offering all pregnant and lactating women lifelong antiretroviral therapy (ART) regardless of the cluster of differentiation 4 (CD4) count and clinical stage [2] to reduce MTCT rate to less than 5% in breastfeeding and less than 2% in non-breastfeeding women [3]. But to achieve this plan, drug adherence is very incredible [4].

Various studies in Africa described different adherence level that is below 95% adherence which not indicates a good level of adherence, in Zambia (82%) [5], Tema center in Ghana (85.1%) [6], and Kenya (89%) [7]. In Ethiopia, three studies conducted in Tigray [8], South wollo [9], and Hadya [10] showed that 12.9%, 12.3%, and 17% of participants don’t have good adherence to option B+ PMTCT respectively. These indicate that still 11%-18% of newborns in probable to be infected by HIV which is far away to reach the goal set globally to end new HIV infection in children by 2030 [11].

Adherence to ART is important to decrease the MTCT rate, prevent the progression of mothers HIV to AIDS, promote viral suppression, and decrease drug resistance [12]. Further, adherence to ART is also important to achieve the goals set globally to ending new HIV/AIDS among children by 2030 [11]. Despite these all importance still adherence to ART is an unsettled problem.

Poor adherence to ART is associated with less effective viral suppression, forgetting when to take the drug [8], living in a rural area, facing challenges on the same day they diagnosed with HIV and initiated lifelong option B+ treatment [9], drug side effects, stigma, and partner/family support [10]. Measuring adherence level and identifying the associated factor is necessary to know the achievement of ART to prevent MTCT.

Maintaining adherence to prescribed ART among pregnant & lactating mothers continues to be a major public health concern in both high-income and low-income countries. To come up with the best outcome, achieving the standard level of drug adherence which is 95% is essential. However, there are few studies conducted in Ethiopia with more focusing on pregnant women while it also essential to include lactating women as they are at risk to transmit HIV to their babies. Therefore, this study aims to assess the level of adherence and factors associated to option B+ PMTCT among HIV+ pregnant and lactating women who are on PMTCT follow-up at the government health facility in Hawassa city, southern Ethiopia.

## Materials and methods

### Study design and setting

An institution-based cross-sectional study was carried out from May 30-June 30/2019 on a sample of 254 HIV+ pregnant and lactating mothers in a governmental health facility of Hawassa city, Southern Ethiopia.

Hawassa is the capital city of SNNPR located 273 km south of Addis Ababa, the capital city of Ethiopia. The city encompasses a total population of 341,659 with a male to female ratio of 51.4% to 48.6% residing in 8 sub-cities in 2016. The city has three governments (two general and another comprehensive) and 4 private primary hospitals, 12 health centers, and many public and private clinics and pharmacies. It has one Government University, many other governments, and private colleges.

### Sample size and sampling procedure

The sample size was determined using the single population proportion formula with considering; 95% confidence interval, 80% power of the test, 5% margin of error, and 81.4% expected level of adherence to option B+ PMTCT in Southern Ethiopia [13]. With a 10% non-response rate, the final sample size was 256. To recruit the calculated sample size, both hospitals (Adare and HUCSR) and three out of 12 health centers (Millennium, Tula, and Adare HC) were purposively selected based on their ART service provision. Then, the proportion to sample size allocation was used to take a sample from each selected health facility. Finally, a simple random sampling technique was carried out to select the allocated participant.

### Data collection tools and procedures

The questionnaire prepared in English was translated into Amharic and translated back into English by a different person to check the consistency in the meaning of the words and concepts. The questionnaires that provide information on socio-demographic characteristics of respondents, patient-related factors, partner/family support-related factors, and health facility-related factors have developed after different literature reviewed. To measure the level of ART adherence, a self-report method included in a multi-method tool to measure ART adherence in the resource-constrained settings was used [14]. The questionnaire was pretested before the actual data collection period to ensure that respondents understand the questions and to check the wording, logic, and the skipping order of the questions, and the necessary amendment was taken. Data collectors and supervisors were trained on the objective, data collection techniques, data quality, and techniques of interview of the study for one day before the actual data collection period. Data was collected by 3 BSc Midwives working outside the study facility and who had previous experience of data collection with PMTCT related studies to minimize social desirability bias. The respondents were interviewed after they attended ANC follow-up visit in a calm private room that was arranged in each study hospital for the data collection purpose. Data consistency and completeness were done daily.

### Statistical analysis

Collected data was cleaned and manually enter into Epidata version 3.1 and exported to SPSS-version 20 statistical software for analysis. Descriptive analysis was done to describe frequency distribution, proportion, the measure of central tendency, and dispersion. Binary logistic regression was done to measure the strength of association between independent and dependent variables using the odds ratio and 95% of confidence interval. Independent variables with P-value <0.25 in binary logistic regression were taken to multiple logistic regression analyses. Then, multiple logistic regression analysis was carried out to identify factors associated with adherence, and P-value <0.05 was considered statistically significant. Finally, the presentation of findings was done using text, frequency tables, graphs, and charts.

### Operationalization of the outcome variable

Adherence: Adherence to ART is ingestion of all anti-retroviral medicines prescribed correctly at the right time.

#### Good adherence

A woman will be considered as good adherence if she responded ‘**No**’ to all (four) questions prepared in the self-report method to assess the adherence level. The self-report questions are:

1. At sometimes, do you find it difficult to remember to take your medication?
2. Do you sometimes take a break from your medication when you feel better?
3. Many patients have trouble with taking their ART doses as prescribed; did you miss any ART doses in the last 3 days?
4. Do you sometimes stop taking medication when you feel worse?

#### Poor adherence

A woman will be considered as poor adherence if she responded ‘**Yes**’ to at least 1 of the four self-report questions [14].

#### Knowledge of PMTCT

Women’s knowledge on PMTCT was categorized as ‘good knowledge’, and ‘poor knowledge’. If they scored mean and above score considered as good knowledge otherwise as poor knowledge [9].

### Ethical consideration

Ethical approval was gained from the Institutional Review Board at the college of medicine and Health Sciences of Hawassa University, Hawassa, Ethiopia. All purposively selected governmental public institutions’ administrative staff and PMTCT workers were informed about the research with a formal letter and ethical approval letter. The study participant was fully informed about the purpose of the study and oral consent was received from each participant before starting an interview.

## Results

### Socio-demographic characteristics

Two hundred fifty-four HIV-positive pregnant and lactating women taking Option B plus ART were included in the study to make a response rate of 99.2%. The mean and standard deviation of the participant’s age was 29.8 (±4.83) years. About 12% weren’t had formal education (Table 1).

**Table 1:** Socio-demographic characteristics of participants among HIV+ pregnant & lactating women in pregnant & lactating mothers in selected governmental health facilities of Hawassa city, Southern Ethiopia, 2019/20 G.C.

Variables	Categories	Number	Percentage (%)
**Age**	≤ 25	46	18.1
	26–30	83	32.7
	≥ 30	125	49.2
**Residence**	Urban	204	80.3
	Rural	50	19.7
**Religion**	Protestant	125	49.2
	Orthodox	105	41.3
	Muslim	24	9.5
**Marital status**	Single	3	1.2
	Married	236	92.9
	Divorced + Widowed	15	5.9
**Educational level**	No formal education	31	12.2
	Grade 1–8	85	33.5
	Grade-12	55	21.7
	College and above	83	32.7
**Participant’s occupation**	Housewife	118	46.4
	Government employee	77	30.3
	Merchant	55	21.7
	Other(s)^a^	4	1.6
**Participant’s partner occupation**	Government employee	92	39
	Merchant	117	49.6
	Farmer	19	8
	Other(s)^b^	8	3.4
**Income(in ETB)**	< 1000	24	9.4
	1000–2000	19	7.5
	2000–3000	29	11.4
	≥3000	182	71.7

Key:

“^a^” shows day laborer (3), private employee (1)

“^b^” shows day laborer (4), private employee (3), and student (1) ETB- stands for Ethiopian Birr.

### Drug and health facility-related characteristics

Among participants, 164 (64.6%) were receiving care at the hospital level. Four in five women, 202 (79.5%) reported that they received counseling about option B plus PMTCT benefits from their service provider. The majority of women, 209 (82.3%) had good knowledge of option B plus PMTCT services (Table 2).

**Table 2:** Health service setting and health care provider relationship with the client among pregnant & lactating mothers in selected governmental health facilities of Hawassa city, Southern Ethiopia, 2019/20 G.C.

Variables	Categories	Number	Percentage (%)
**Place of service**	Health center	90	35.4
	Hospital	164	64.6
**Counseling**	No	52	20.5
	Yes	202	79.5
**Think as data is kept confidential**	No	67	26.4
	Yes	187	73.6
**Area is suitable for receiving care**	No	6	2.4
	Yes	248	97.6
**Relation with HP**	Poor	19	7.5
	Good	235	92.5
**Time to reach HF**	≤ 1 hour	185	72.8
	> 1 hour	69	27.2
**Knowledge on option B plus PMTCT**	Poor	45	17.7
	Good	209	82.3

HP-stands for a health care provider, HF-stands for health facility, PMTCT-stands for prevention of mother to child transmission.

### Patient-related factors

**Fig 1:**
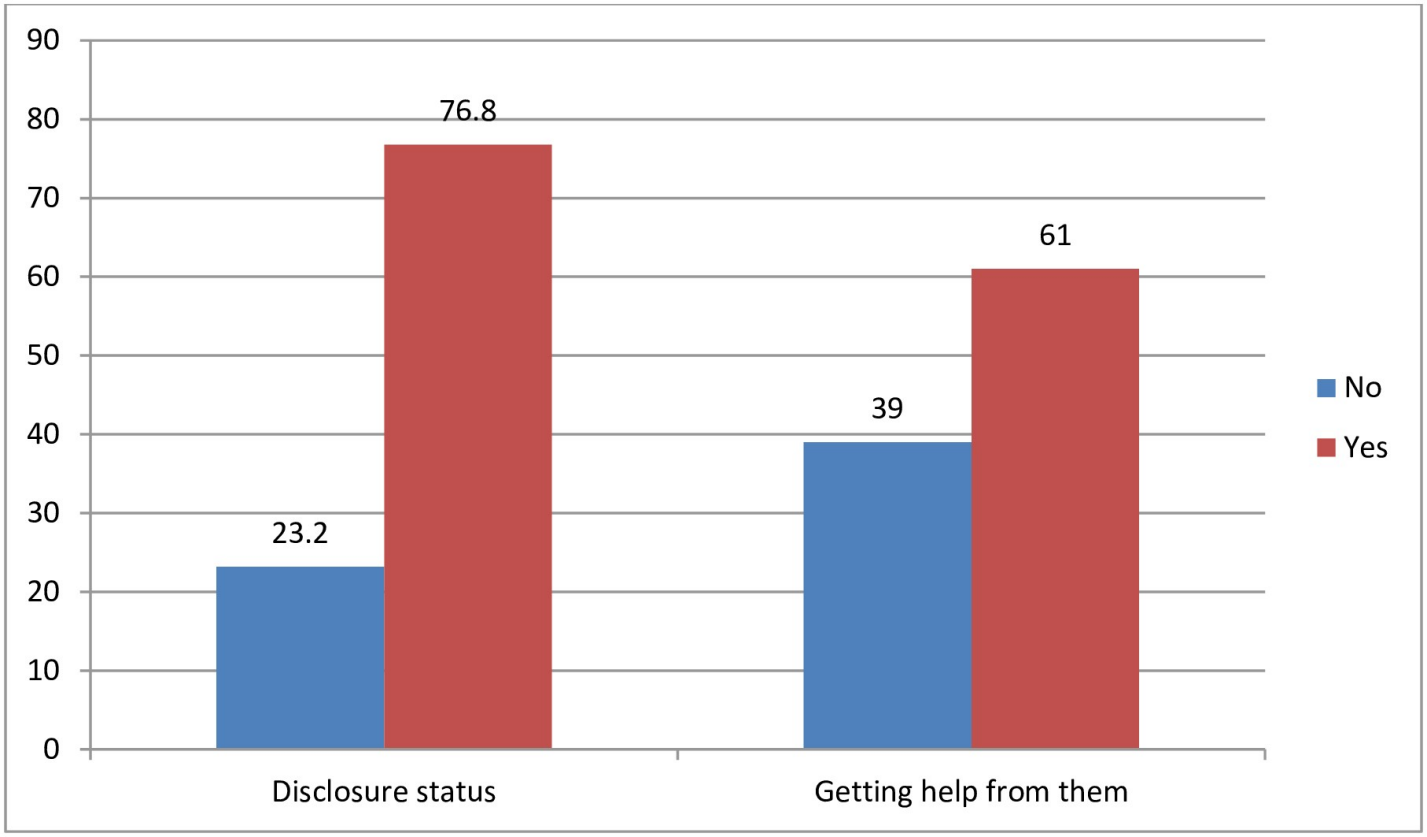
Percentage of disclosure of their status and getting support from them among pregnant & lactating mothers in selected governmental health facilities of Hawassa city, Southern Ethiopia, 2019/20 GC.

Three in four participants, 195 (76.8%) disclosed HIV status to their partners, and 155 (61%) reported that they received support from their partner to take their drug as prescribed (Fig 1).

### Adherence level of option B plus PMTCT service

**Fig 2:**
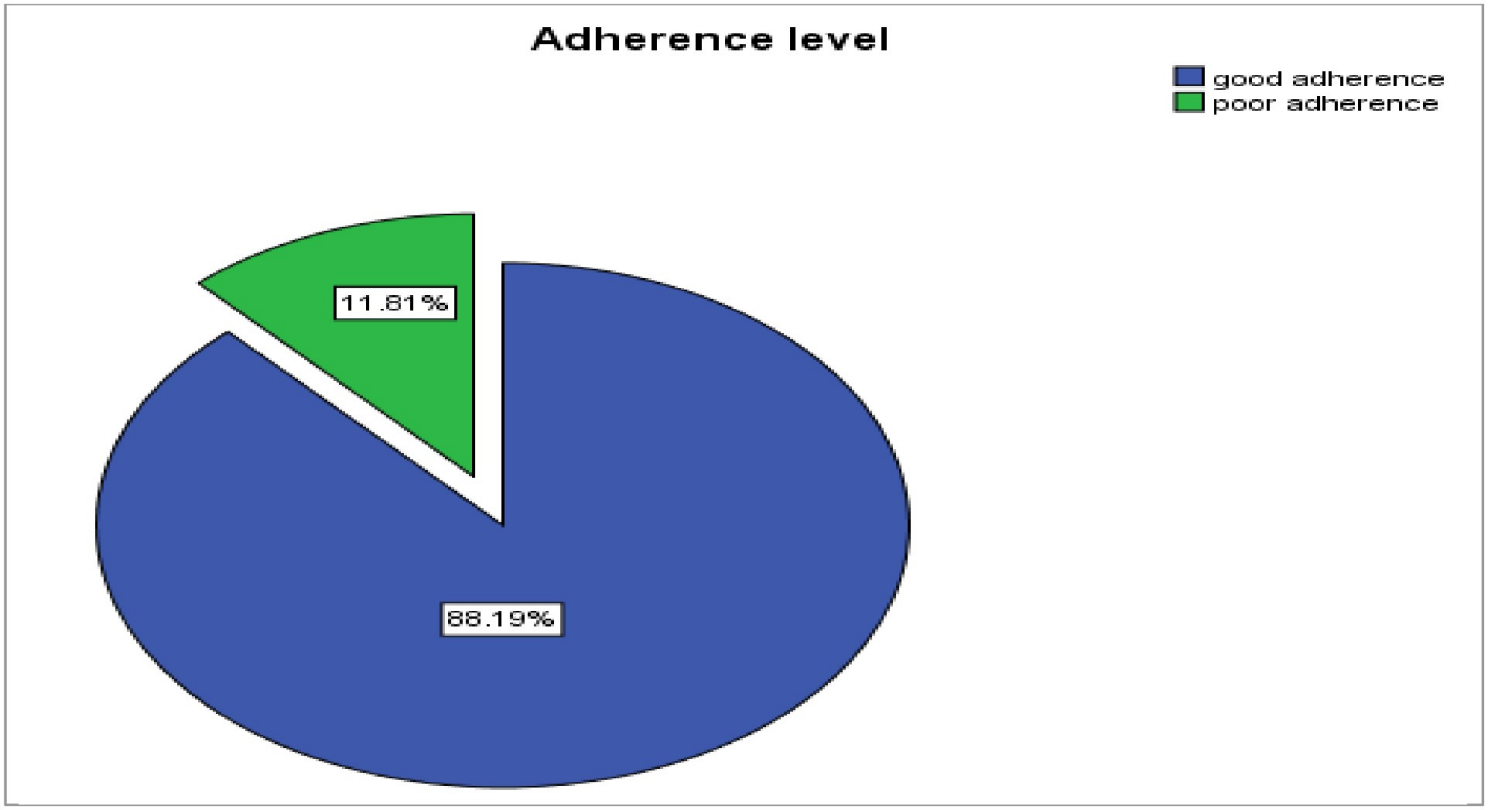
Adherence level of option B plus PMTCT service among pregnant & lactating mothers in selected governmental health facilities of Hawassa city, Southern Ethiopia, 2019/20 GC.

Overall, 88.2% (95% CI: 84.3, 92.1) of the respondents were adherent to Option B+ PMTCT drugs (Fig 2). The frequently mentioned reasons for non-adherence were difficult to remember the time for taking drugs. This study showed that 30 (11.8%) of the respondents were non-adherent to Option B+ PMTCT care and support. Out of these non-adherent respondents, 10 (33.3%) had missed their ARV medication within the last three days before the study. Among the reasons to miss their Option B+ PMTCT ART, difficult to remember to take medications (52%) was the dominant obstacle for adherence followed by stopping taking drugs while felt better (Table 3).

**Table 3:** The adherence questions and the answers given among pregnant & lactating mothers in selected governmental health facilities of Hawassa city, Southern Ethiopia, 2019/20 G.C.

Variables	Categories	Number	Percentage (%)
Difficult to remember to take a drug	Yes	26	10.2
	No	228	89.8
Stop taking medicine while feeling better	Yes	9	3.5
	No	245	96.5
Did you miss any ART dose in the last 03 days	Yes	10	3.9
	No	244	96.1
Stop taking medicine while feeling worse	Yes	5	2.0
	No	249	98.0

### Factors associated with adherence level

The multivariable logistic regression analysis result showed that age of participants, educational level, Knowledge of PMTCT, getting help from partner/family, and experiencing side effects had a significant association with option B plus PMTCT adherence level (Table 4).

**Table 4:** Bivariate and multivariate analysis result of factors associated with adherence level of option B + PMTCT among pregnant & lactating mothers in selected governmental health facilities of Hawassa city, Southern Ethiopia, 2019/20 G.C.

Variables	Adherence level		COR, 95% CI	AOR, 95% CI
	Good Number (%)	Poor Number (%)		
**Age**				
**≤ 25**	**32(69.6)**	**14(30.4)**	**0.20 (0.08, 0.49)**	0.12 (0.03, 0.42)**
**25–30**	77(92.8)	6(7.2)	1.12 (0.39, 3.2)	0.9 (0.24, 3.32)
**≥ 30**	**115(92)**	**10(8)**	**ref**	**ref**
**Educational level**				
**No formal education**	**21(67.7)**	**10(32.3)**	**0.135 (0.04, 0.44)**	0.12 0(.03, 0.51)**
**Grade 1–8**	77(90.6)	8(9.4)	0.62 (0.19, 1.97)	1.06 (0.26, 4.3)
**Grade 9–12**	48(87.3)	7(12.7)	0.44 (0.13, 1.46)	1.08 (0.25, 4.6)
**College and above**	**78(94)**	**5(6)**	**ref**	**ref**
Time option B+ started				
During this pregnancy	95(89.6)	11(10.4)	1.02 (0.44, 2.4)	3.6 (0.43, 29.4)
During breastfeeding	19(76)	6(24)	0.37 (0.13, 1.1)	1.74 (0.2, 15.5)
Previously known	110(89.4)	13(10.6)	ref	ref
Disclosure status				
No	47(79.7)	12(20.3)	ref	ref
Yes	177(90.8)	18(9.2)	2.5 (1.13, 5.6)	1.08 (0.33, 3.6)
**Knowledge on PMTCT**				
**Poor**	**34(75.6)**	**11(24.4)**	**ref**	**ref**
**Good**	**190(90.9)**	**19(9.1)**	**3.24 (1.41, 7.4)**	3.6 (1.12, 11.3)*
Relationship with HP				
Poor	13(68.4)	6(31.6)	ref	ref
Good	211(89.8)	24(10.2)	4.1 (1.41, 11.7)	3.2 (0.7, 14.9)
**Getting help from other**				
**No**	**80(95.9)**	**19(4.1)**	**ref**	**ref**
**Yes**	**144(42.1)**	**11(57.9)**	**3.1(1.4, 6.9)**	4.5 (1.6, 12.4)**
**Experiencing a side effect**				
**No**	**185(94.4)**	**11(5.6)**	**ref**	**ref**
**Yes**	**39(67.2)**	**19(32.8)**	**0.122 (0.05, 0.28)**	0.11 (0.04, 0.32)***
Counsel				
No	39(74)	13(26)	ref	ref
Yes	185(91.7)	17(8.3)	3.63 (1.63, 8.1)	2.64 (0.93, 7.5)

Note:

*represents P<0.05,

**P≤0.01,

***P≤0.001.

COR stands for Crude odd Ratio, AOR stands for Adjusted odd Ratio, PMTCT stands for Prevention of mother to child transmission, and HP stands for Health care Provider.

Those participants who were in the ≤ 25 age group were 88% less likely to adhere to option B plus PMTCT care and services when compared to the ≥ 30 age group with [AOR = 0.12, 95% CI (0.03, 0.42)]. Among participants, those with no formal educational level were about 88% less likely to be adhered to compared to those with college and above. [AOR = 0.12, 95% CI (0.03, 0.51)].

In this study, women who have good knowledge on option B plus PMTCT were 3 times more likely to adhere when compared to their counterparts [AOR = 3.6, 95% CI (1.16, 11.3)]. Women who get support from their supporters (to whom they disclosed their status) were 4 times more likely to adhere than their counterparts [AOR = 4.5, 95% CI (1.62, 12.4)]. Experiencing drug side effects also had a significant association with adherence level. Those who experienced side effects were 89% less likely to adhere when compared to those who didn’t experience [AOR = 0.114, 95% CI (0.04, 0.32)].

## Discussion

In this study, the adherence level of option B plus PMTCT and associated factors among HIV-positive pregnant and lactating women were identified. The overall adherence level is 88.2% which suboptimal when compared to the recommended adherence level of 95% and above to prevent vertical transmission of HIV and drug resistance [4]. The associated factors were related to the age of participants, educational level, Knowledge of PMTCT, getting help from partner/family, and experiencing a side effect.

The overall adherence level to option B+ PMTCT drugs among HIV-positive pregnant and lactating women was 88.2%. This finding is consistent with studies done in, Tigray (87.1%) and south wollo (87.9%) [8,9].

On contrary, the adherence level in this study is higher than studies conducted in different places like 81.4% in southern Ethiopia [13], 82.2% in east shewa, Ethiopia [15], 83.7% in Hadya, [10], and 82.5% in Zambia [5]. This discrepancy might be accountable for the population and study design used, in addition to others like infrastructure, awareness, time of studies. Studies conducted in southern Ethiopia, east shewa, and Hadya didn’t include lactating mothers as this might be the reason why adherence in this study is higher. For a study in Zambia, the study design used which is a prospective cohort might be matter.

However, the level of adherence of this study is slightly lower than a study conducted in Ukraine 92% for postpartum mothers [16]. This discrepancy might be due to the difference in population and setting in which the study was conducted.

Those participants who were in ≤ the 25 age group were 88% less likely to adhere to option B plus PMTCT care and services when compared to ≥ the 30 age group with [AOR = 0.12, 95% CI (0.03, 0.42)]. This finding is similar to a study conducted in Kenya which stated that odds of starting and continuing ART were higher as age increase [7]. This might be due to the responsibility and accountability the older ages take to care of children and family which make them take their drug as prescribed to become healthier.

In this study educational status was strongly associated with PMTCT adherence. Respondents with no formal educational level were about 88% less likely to be adhered to compared to those with college and above [AOR = 0.12, 95% CI (0.03, 0.51)]. The finding is in line with studies done in East shewa, Ethiopia [15], Kenya, and Ghana [7,17]. This might be due to the reality that better-educated people have access to information which in turn increases their healthcare-seeking behavior.

Those women who have good knowledge of option B plus PMTCT were 3 times more likely to have adhered when compared to their counterparts [AOR = 3.6, 95% CI (1.16, 11.3)]. This finding is consistent with the finding of one systematic review in Sub-Saharan Africa and another study conducted in Ghana which stated that poor knowledge of PMTCT was found as the main reason to quit PMTCT service and be less adhered to ART [17,18]. This is might be due to knowing the benefit and effectiveness of taking ART drugs as prescribed.

Our study showed that women who received support from their partner/family (to whom they disclosed their status) were 4 times more likely to have adhered than their counterparts [AOR = 4.5, 95% CI (1.62, 12.4)]. This finding is similar to a study conducted in Hadya which reported that women with good partner involvement in PMTCT care and support had 72% more likely to be adherent to option B+ PMTCT as compared to low [10]. This might be due to the usual benefit of receiving supports for moral encouragement and health care assistance through reminding the time of drug-taking.

Adherence to option B+ was also strongly associated with the drug side effect. Those who experienced side effects were 89% less likely to have adhered when compared to those who didn’t experience [AOR = 0.114, 95% CI (0.04, 0.32)]. This finding is in line with the studies conducted in East shewa, Ethiopia, and Ukraine [15,16]. This might be due to feeling discomfort which leads to reducing their courage to take drugs as prescribed.

This study revealed that getting counseled is not significantly associated with adherence to option B+ which is in line with the study done in Southern Ethiopia [13]. However, receiving counsel on the importance of taking the drug as prescribed and its side effect was significantly associated with good adherence in different studies [8,10,15]. This difference might be due to the setting and time at which studies were conducted.

### Limitation of the study

In this study, the adherence level was measured using participants’ self-report method as a result the adherence category may be subjected to recall bias and misclassification bias which in turn overestimate the adherence level.

## Conclusion

The level of adherence to option B+ PMTCT drug was 88.2% which is suboptimal. Ages, educational level, knowledge on PMTCT, getting support from partner/family, and drug side effect were significantly associated with adherence. Therefore, educating and counseling on the service of PMTCT to improve their knowledge and encouraging partner/family involvement in care are mandatory to achieve the standard adherence level.

## Supporting information

S1 Data (SAV)

## References

[1] Nations U. Global HIV & AIDS statistics—2018 fact sheet. UNAIDS org. 2018.

[2] UNICEF. Options B and B+: Key considerations for countries to implement an equity-focused approach. Eliminating new HIV infections among children and keeping mothers living with HIV alive and well. 2013.

[3] UNAIDS U. Countdown to ZERO: global plan towards the elimination of new HIV infections among children by 2015 and keeping their mother alive. UNAIDS; 2011.

[4] World Health Organization W. Use of antiretroviral drugs for treating pregnant women and preventing HIV infection in infants. WHO, Geneva, April. 2012. 26180894

[5] Okawa S, Chirwa M, Ishikawa N, Kapyata H, Msiska CY, Syakantu G, et al. Longitudinal adherence to antiretroviral drugs for preventing mother-to-child transmission of HIV in Zambia. BMC pregnancy and childbirth. 2015;15(1):258. doi: 10.1186/s12884-015-0697-7 26459335 PMC4603915

[6] Awittor RJE. Adherence to Antiretroviral Therapy (ART) Among HIV Positive Women at Antiretroviral Centers in Tema: University of Ghana; 2012.

[7] Ayuo P, Musick B, Liu H, Braitstein P, Nyandiko W, Otieno-Nyunya B, et al. Frequency and factors associated with adherence to and completion of combination antiretroviral therapy for prevention of mother to child transmission in western Kenya. Journal of the International AIDS Society. 2013;16(1):17994. 23336727 10.7448/IAS.16.1.17994 PMC3536941

[8] Ebuy H, Yebyo H, Alemayehu M. Level of adherence and predictors of adherence to the Option B+ PMTCT program in Tigray, northern Ethiopia. International Journal of Infectious Diseases. 2015;33:123–9. doi: 10.1016/j.ijid.2014.12.026 25529555

[9] Tsegaye D, Deribe L, Wodajo S. Levels of adherence and factors associated with adherence to option B+ prevention of mother-to-child transmission among pregnant and lactating mothers in selected government health facilities of South Wollo Zone, Amhara Region, northeast Ethiopia, 2016. Epidemiology and health. 2016;38. 10.4178/epih.e2016043 PMC5177807 27733034

[10] Lodebo TM, Suloro JA. Level of Adherence and Associated Factors to Option B+ PMTCT among HIV Positive Pregnant Women in Hadiya Zone, Southern Ethiopia. Global Journal of Health Sciences. 2017;2(1):39–58.

[11] Joint United Nations HA. Fast-track: ending the AIDS epidemic by 2030. Geneva: UNAIDS. 2014.

[12] Paterson DL, Swindells S, Mohr J, Brester M, Vergis EN, Squier C, et al. Adherence to protease inhibitor therapy and outcomes in patients with HIV infection. Annals of internal medicine. 2000;133(1):21–30. doi: 10.7326/0003-4819-133-1-200007040-00004 10877736

[13] Tesfaye DJ, Hibistu DT, Abebo TA, Asfaw FT, Lukas K, Laelago T, et al. Option B plus antiretroviral therapy adherence and associated factors among HIV positive pregnant women in Southern Ethiopia. BMC pregnancy and childbirth. 2019;19(1):82. doi: 10.1186/s12884-019-2228-4 30819147 PMC6394094

[14] Steel G, Nwokike J, Joshi MP. Development of a multi-method tool to measure ART adherence in resource-constrained settings: the South Africa experience. RPM Plus. 2007;6.

[15] Lencha MT. Adherence to Option B+ and Associated Factors Among Pregnant Women on PMTCT Services at Public Health Facilities of East Shawa Zone, Oromia, Ethiopia. Women’s Health and Reproductive Medicine. 2018;2. 10.1016/j.srhc.2019.100459 31442747

[16] Bailey H, Thorne C, Malyuta R, Townsend CL, Semenenko I, Cortina-Borja M, et al. Adherence to antiretroviral therapy during pregnancy and the first year postpartum among HIV-positive women in Ukraine. BMC Public Health. 2014;14(1):993. doi: 10.1186/1471-2458-14-993 25248469 PMC4180980

[17] Boateng D, Kwapong GD, Agyei-Baffour P. Knowledge, perception about antiretroviral therapy (ART) and prevention of mother-to-child-transmission (PMTCT) and adherence to ART among HIV positive women in the Ashanti Region, Ghana: a cross-sectional study. BMC women’s health. 2013;13(1):2. 23336813 10.1186/1472-6874-13-2 PMC3563602

[18] Gourlay A, Birdthistle I, Mburu G, Iorpenda K, Wringe A. Barriers and facilitating factors to the uptake of antiretroviral drugs for prevention of mother-to-child transmission of HIV in sub-Saharan Africa: a systematic review. Journal of the International AIDS Society. 2013;16(1):18588. doi: 10.7448/IAS.16.1.18588 23870277 PMC3717402

